# Efficacy of voriconazole combined with caspofungin in treating Chronic Obstructive Pulmonary Disease patients with invasive pulmonary aspergillosis: impacts on clinical outcomes, inflammatory cytokines, and immune markers

**DOI:** 10.3389/fimmu.2026.1831585

**Published:** 2026-07-21

**Authors:** Jianfeng Zhang, Zhiyi Huang

**Affiliations:** 1 Emergency Department, Jinhua People’s Hospital, Jinhua, Zhejiang, China; 2 General Medicine Department, Yiwu Hospital of Integrated Traditional Chinese and Western Medicine, Yiwu, Zhejiang, China

**Keywords:** caspofungin, combination therapy, COPD, invasive pulmonary aspergillosis, voriconazole

## Abstract

**Objective:**

To compare the efficacy and safety of voriconazole plus caspofungin versus voriconazole alone in COPD patients with invasive pulmonary aspergillosis (IPA) and to determine whether the combination accelerates symptom resolution, reduces fungal and inflammatory load, and improves lung function and quality of life.

**Methodology:**

We retrospectively analyzed 152 COPD-IPA patients treated between April 2022 and April 2024, divided into an observation group (voriconazole + caspofungin, n=76) and control group (voriconazole alone, n=76). Outcomes included clinical efficacy, symptom resolution, inflammatory markers, and safety.

**Results:**

The observation group demonstrated superior outcomes including higher total efficacy (89.47% vs. 65.79%, P<0.05), faster symptom relief (cough: 5.36 vs. 7.89 days; fever: 7.06 vs. 9.44 days; P<0.05), and shorter hospitalization (12.28 vs. 15.62 days, P<0.05). Post-treatment analysis revealed lower β-D-glucan levels (15.64 vs. 20.16 pg/mL), reduced inflammatory markers (CRP, IL-12, IL-15, IL-17, TNF-α; P<0.05), and improved lung function (FEV1/FVC: 57.56% vs. 54.02%, P<0.05). Quality of life scores significantly improved (P<0.05) with comparable adverse events (7.89% vs. 13.16%, P>0.05).

**Conclusion:**

Voriconazole combined with caspofungin in oral sequential treatment of COPD combined with IPA can accelerate the relief of clinical symptoms and signs of patients, inhibit pathogenic bacteria and inflammatory response, improve lung function and quality of life of patients, improve efficacy, safety and reliability.

## 1 Introduction

Chronic Obstructive Pulmonary Disease (COPD) is a prevalent respiratory disorder characterized by progressive airflow limitation and chronic inflammation, leading to debilitating symptoms such as dyspnea and recurrent infections (1). The pathophysiology of COPD involves impaired mucociliary clearance, epithelial damage, and dysregulated immune responses, creating a microenvironment conducive to fungal colonization (2). Notably, the escalating use of broad-spectrum antibiotics, glucocorticoids, and invasive procedures in COPD management further disrupts host defenses, significantly increasing the risk of invasive pulmonary aspergillosis (IPA) (3). Research indicates that following malignancies and organ transplantation, COPD ranks as the third leading predisposing condition for developing IPA. COPD-associated IPA is particularly challenging due to overlapping clinical features with routine exacerbations, often delaying diagnosis and treatment (4).

IPA in COPD patients is driven by Aspergillus species exploiting compromised airway barriers and immune dysfunction. Alveolar macrophages, the first line of defense against inhaled spores, exhibit reduced phagocytic capacity in COPD, while glucocorticoid therapy suppresses Th1-mediated immunity, shifting the balance toward Th17-driven inflammation (5). This cytokine imbalance perpetuates tissue damage and impairs fungal clearance, contributing to poor outcomes. Current guidelines recommend voriconazole as first-line therapy for IPA due to its broad-spectrum activity and high oral bioavailability (6). However, monotherapy failures remain common, attributed to pharmacokinetic variability, drug interactions, and resistance mechanisms in Aspergillus (7).

Caspofungin, an echinocandin, complements voriconazole by targeting β-(1,3)-D-glucan synthase, a mechanism distinct from azoles. Synergistic effects have been reported in refractory cases, but clinical evidence in COPD-IPA populations is limited (8). Additionally, the impact of combination therapy on immune modulation—specifically, IL-12 (Th1), IL-15 (NK/T-cell activation), and IL-17 (Th17)—remains unexplored in this context. To address these uncertainties, this retrospective investigation enrolled 152 COPD patients with concurrent IPA to evaluate whether voriconazole–caspofungin combination therapy improves clinical outcomes, accelerates cytokine normalization, and modulates immune markers compared to voriconazole alone.

## 2 Data and methods

**Figure 1:**
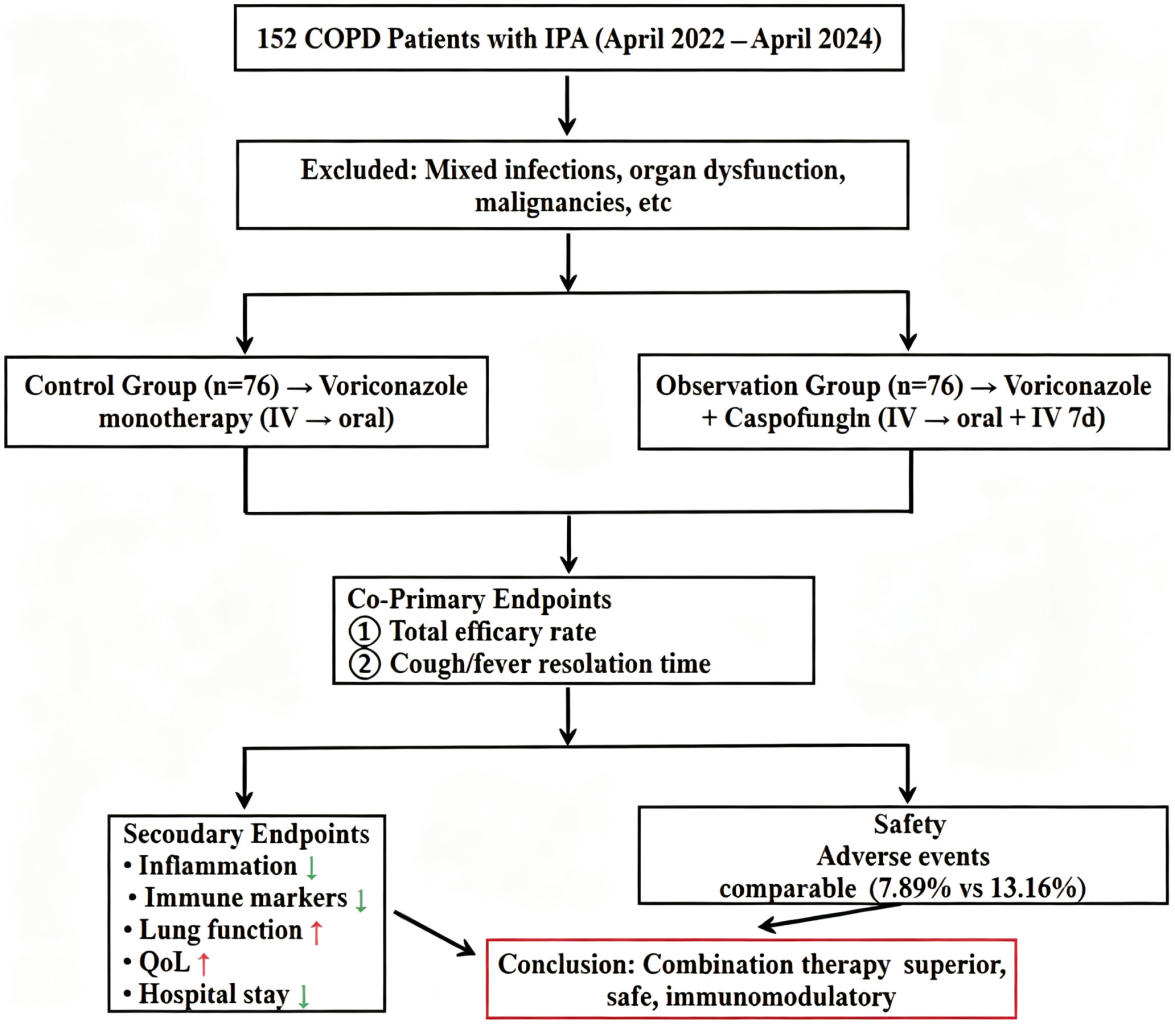
Schematic summary of study design and key findings.

The overall study design, patient selection, treatment allocation, outcome assessments, and major findings are summarized in **Figure 1**.

### 2.1 General clinical data

This study examined 152 COPD patients with concurrent IPA who received treatment at our medical center between April 2022 and April 2024. All eligible patients meeting inclusion criteria (n=152) during the study period were included to maximize power. BALF cultures/PCR and serology confirmed no concurrent pathogens. Sample size was estimated using GPower 3.1, including primary outcome: difference in total efficacy rate (65.8% vs. 89.5%); effect size: 23.7% (α=0.05, power=80%, two-tailed test); attrition rate: 10%, yielding a target of 76/group. Subjects were equally allocated into two cohorts (n=76 each) based on therapeutic interventions. Patients were allocated to treatment groups based on clinician discretion during hospitalization. To minimize confounding, we ensured baseline equivalence in demographics, disease severity, and comorbidities. Multivariate logistic regression was used to adjust for covariates influencing outcomes. The gender, age, course of COPD, body mass index, gender, course of IPA, drinking history, smoking history, COPD grade, and comorbidities were comparable in the two groups *(P>0.05*), see **Table 1**. This study was approved by the Ethics Committee of Jinhua People’s Hospital.

**Table 1:** Comparison of the clinical data of the two groups.

Data	Observation group (n=76)	Control group (n=76)	*t*/*χ^2^*	*P*
Age(year)	45.86 ± 6.82	46.04 ± 6.91	0.162	0.782
Course of COPD(year)	5.66 ± 1.03	5.58 ± 0.97	0.493	0.623
BMI(kg/m^2^)	22.34 ± 2.11	22.28 ± 2.06	0.177	0.859
Gender(Male/Female)	40/36	46/30	0.482	0.488
Course of IPA(d)	4.11 ± 1.25	4.06 ± 1.14	0.258	0.797
Drinking history	32(42.11)	40(52.63)	0.844	0.358
Smoking history	28(36.84)	24(31.58)	0.234	0.629
COPD grade				
I	8(10.53)	10(13.16)	0.902	0.825
II	24(31.58)	30(39.47)		
III	38(50.00)	32(42.11)		
IV	6(7.89)	4(5.26)		
Complication				
Hyperlipidemia	14(18.42)	20(26.32)	0.382	0.409
Diabetes	12(15.79)	18(23.68)	0.748	0.387
Heart disease	8(10.53)	4(5.26)	0.181	0.671
Hypertension	6(7.89)	2(2.63)	0.264	0.608

### 2.2 Inclusion and exclusion criteria

(1) Eligible participants met the following requirements: adult status (≥18 years), confirmed diagnoses of COPD (COPD was diagnosed per GOLD 2023 guidelines: post-bronchodilator FEV1/FVC <0.70 with compatible symptoms. Severity was staged I–IV based on FEV1% predicted and exacerbation history) (9) and IPA (IPA was confirmed using modified EORTC/MSG criteria, BALF GM index ≥1.0 or serum ≥0.5, BALF culture/PCR positive for Aspergillus, nodules, cavities) (10); Good adherence (Pill counts: ≥80% of prescribed doses returned at follow-up; No missed doses recorded in medical charts; Structured interviews confirming compliance); No history of relevant drug allergies; No history of relevant drug use before enrollment; No autoimmune disease; Can tolerate bronchoalveolar lavage. (2) Exclusion criteria: major organ dysfunction (Renal: eGFR <30, hepatic: Child-Pugh ≥B, cardiac: NYHA III–IV), psychiatric disorders, malignancies, interstitial pneumonia, concurrent infections, or withdrew consent.

### 2.3 Methods

#### 2.3.1 Basic treatment

According to their conditions, both groups were given symptomatic treatments such as oxygen inhalation, correction of electrolyte imbalance, and maintenance of acid-base balance.

#### 2.3.2 Control group

Voriconazole is given intravenous/oral sequential therapy. Voriconazole injection (Livzon Pharmaceutical Factory of Livzon Group, Guoyao Zhunzi H20064493) 6 mg/kg on the first day, intravenous drip, administered once in 12 h, 4 mg/kg on the second day, administered once in 12 h, intravenously administered until symptom relief, body temperature control after oral administration, voriconazole capsule (Deyang Huakang Pharmaceutical Co., Ltd., Guoyao Zhunzi H20080787), 200 mg/time, 12 h/time, a total of 14 days of treatment.

#### 2.3.3 Observation group

In addition to control group therapy, patients received caspofungin (Labora, imported drug registration certificate number H20080617) through intravenous administration. The initial dose was 70 mg administered once daily on day one, followed by 50 mg once daily starting on day two. This regimen continued for a 7-day period (11).

#### 2.3.4 Criteria of efficiency

Cure: Symptoms such as fever and sputum production disappear, lung imaging examination returns to normal, and etiological examination turns negative. Obvious treatment effect: The clinical symptoms were significantly relieved, and the lung imaging was significantly improved by >80%. Effective: The clinical symptoms and the lung imaging are improved by 50%~80%. Ineffective: Those who do not meet the above criteria. Total effective rate = (cure + Obvious treatment effect + effective)/total number of cases× 100%.

#### 2.3.5 Specimen collection and detection

1. Main reagents and instruments: interleukin-12, IL-15, IL-17, Tumor Necrosis Factor-α (TNF-α) kit (Shanghai Xinfan Bio); GKT-5M Set Dynamic Fungus Detection Kit (Beijing Jinshanchuan Technology Development Co., Ltd.); Platelia Aspergillus kit (Bio-Rad, France); MB-80 microbial dynamic detection system (Beijing Jinshanchuan); Pentraxin 3 (PTX3) kit (Shanghai Xinyu Biotechnology Co., Ltd.); Toll-like receptor 2 (TLR2) in peripheral blood (Shanghai Hengyuan Biochemical Reagent Co., Ltd.).
2. Specimen collection: a) Blood serum procurement: Venous blood (2ml) was drawn from fasting subjects prior to and following therapeutic intervention. After allowing coagulation, samples underwent centrifugation for 10 minutes. The resulting supernatant was extracted and preserved for subsequent analysis. b) Bronchoalveolar lavage collection: Before and after treatment, under the direct view of the bronchoscopic lens, the lesion lung segment is lavaged in stages, the lavage volume is 1ml/kg, and the total amount is less than 20ml/time, the lavage solution should be aspirated at 13.3~20kPa negative pressure, and the alveolar lavage solution should be collected and sent for testing.
3. Detection method: G test was used to detect the concentration of 1,3-β-D glucan (BG), and appropriate samples were taken for Galactomannan (GM) test, and the absorbance of recorded samples/absorbance of standard serum (I value) was detected.
4. Peripheral blood CRP and WBC measurements were conducted using an URIT-8280 automatic biochemical analyzer. Bronchial lavage fluid cytokines (IL-12, IL-15, IL-17 and TNF-α) were quantified via enzyme-linked immunosorbent assay techniques. Similarly, serum levels of PTX3 and TNF-α underwent evaluation through immunosorbent assay methodology.
5. A GEM 4000 plus blood gas analyzer facilitated measurement of arterial gas parameters, including oxygen partial pressure (PaO_2_), carbon dioxide tension (PaCO_2_), and oxygen saturation (SaO_2_). Pulmonary function parameters—FEV1 (forced expiratory volume in the first second), FVC (forced vital capacity), and their ratio (FEV1/FVC%)—were assessed using Jaeger pulmonary function equipment manufactured in Germany.
6. qPCR: The relative expression of TLR2 and PTX3 mRNA were detected by qPCR. Collect 2 mL of fasting venous blood, centrifuge at 3,000 rpm for 10 min, and store serum at -80 °C. Use TRIzol or a commercial kit (Qiagen RNeasy). Reverse transcribe 1 μg RNA using PrimeScript RT Kit (Takara) with random hexamers. Primers: TLR2:F: 5’-GGCGTTCTCTCAGGTGACTG-3’, R: 5’-CCCTGTCTTCCTGCCTTCAC-3’. PTX3: F: 5’-GCTCTCTGGTCTGCAGTGTT-3’, R: 5’-CTTGTCCCATTCCGAGTGCT-3’. GAPDH: F: 5’-AATGGGCAGCCGTTAGGAAA-3’, R: 5’-GCGCCCAATACGACCAAATC-3’. Conditions: 95 °C for 30 sec; 40 cycles of 95 °C (5 sec) and 60 °C (30 sec). Calculate relative expression (2−ΔΔCt method) using GAPDH for normalization.
7. Quality of life assessment: Patient well-being was evaluated via SGRQ (St. George’s Respiratory Questionnaire), which encompasses three dimensions. Each dimension ranges from 0 to 100 points, with lower scores indicating superior life quality. Clinical symptom evaluation: evaluated by COPD scale (CAT), the score range is 0~40 points, the lower the score, the milder the patient’s symptoms.
8. Adverse reactions: Adverse events were categorized per CTCAE v5.0. Liver impairment required ALT/AST >3× ULN; visual abnormalities triggered ophthalmologic referral if unresolved at 48 hours. Kidney function was not routinely monitored per institutional protocols for these antifungals. Systemic aspergillosis was excluded via serum GM (< 0.5), blood cultures, and abdominal imaging. Adverse events were attributed to drugs only after ruling out fungal progression (repeat GM/BG, imaging).

### 2.4 Outcome measures

Two co–primary endpoints were prespecified: (1) total treatment efficacy rate (composite of cure, obvious improvement, and effective), and (2) time to clinical response, defined as days to cough relief and fever resolution. All other outcomes were secondary and included the following measures: (a) bronchoalveolar lavage fluid biomarkers (BG concentration and I value); (b) time to pulmonary sign resolution and length of hospital stay; (c) serum CRP and WBC, as well as bronchoalveolar lavage fluid cytokines (IL–12, IL–15, IL–17, TNF–α); (d) serum immune markers (TLR2 mRNA and PTX3); (e) arterial blood gas parameters (PaO_2_, PaCO_2_, SaO_2_); (f) pulmonary function indices (FEV_1_, FVC, FEV_1_/FVC); (g) quality of life (SGRQ) and symptom severity (CAT) scores; and (h) adverse event rates. All comparisons were conducted between groups at baseline and after treatment, as detailed in the statistical analysis section.

### 2.5 Statistical analysis

Data were analyzed using SPSS 22.0. Continuous variables were expressed as mean ± SD after confirming normality with Shapiro-Wilk tests (P>0.10 for parametric tests). Independent-samples t-tests were used for between-group comparisons, and paired-samples t-tests for within-group comparisons (before vs. after treatment), as all continuous data were normally distributed (Shapiro-Wilk P>0.10). Chi-square tests for categorical variables. Primary outcomes (total efficacy rate, time to clinical response) used Bonferroni correction (α=0.025). Secondary outcomes applied Benjamini-Hochberg FDR control (q<0.05). The statistical analysis adjusted for predefined covariates including age, smoking history (pack-years), baseline PaO_2_, COPD severity (GOLD stage), BMI, comorbidities (diabetes, hypertension, hyperlipidemia), and concurrent glucocorticoid use, using backward elimination (retention P<0.05) with all variance inflation factors <3.

Multivariate logistic regression was used to identify factors associated with lung function improvement (≥5% increase in FEV_1_/FVC). Covariates included age, baseline PaO_2_, COPD grade, smoking history, and treatment group, with backward elimination (retention P<0.05).

## 3 Results

### 3.1 Clinical efficacy

**Figure 2:**
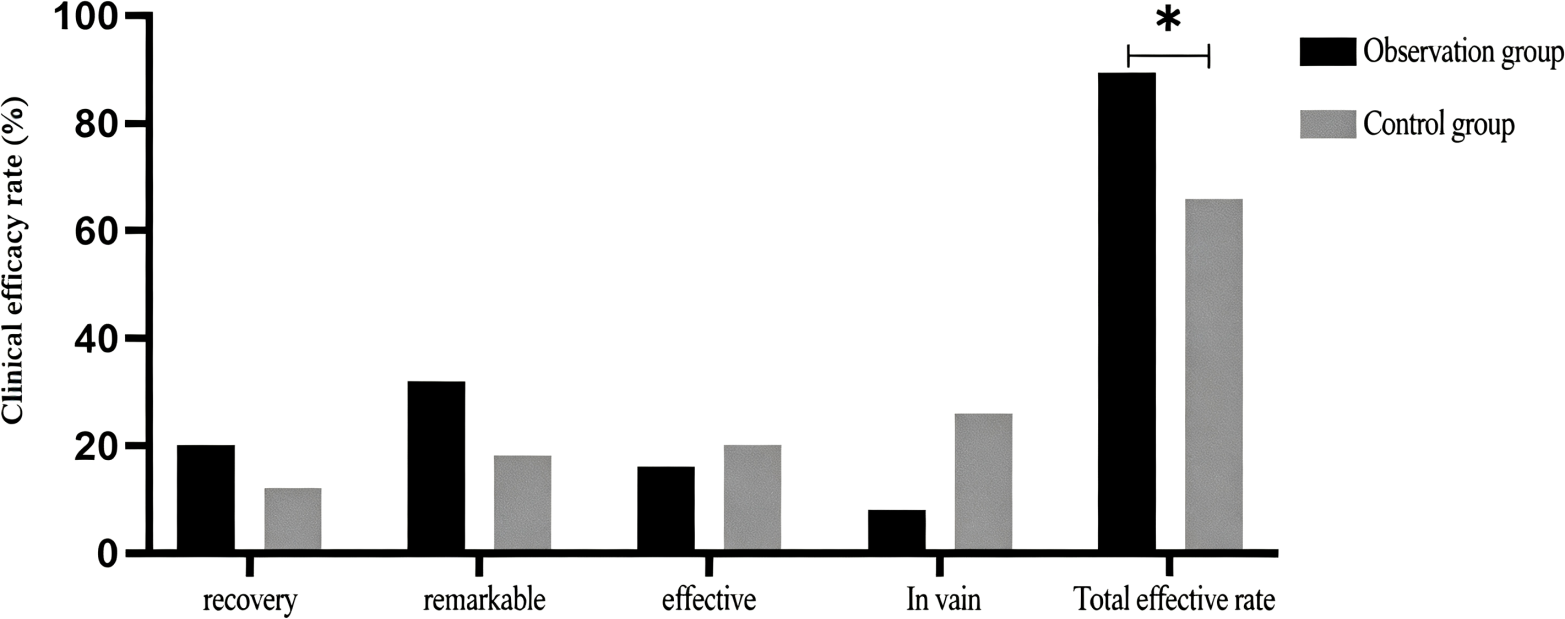
Comparison of clinical efficacy of patients (^*^P<0.05).

The observation group (voriconazole + caspofungin) demonstrated a significantly higher total effective rate (89.47% vs. 65.79%, χ^2^ = 8.102, P = 0.004, **Table 2**, **Figure 2**). The number needed to treat (NNT) was 5, indicating that for every five patients treated with combination therapy, one additional patient achieved clinical improvement. The 34.21% inefficacy rate in the control group (P = 0.004) may reflect limitations of monotherapy, such as pharmacokinetic variability or emerging azole resistance in Aspergillus species.

**Table 2:** Comparison of the efficacy of the two groups *n* (%).

Group	n	Cure	Obvious treatment effect	Effective	Ineffective	Total effectiveness	Risk difference (95% CI)	RR (95% CI)	NNT (95% CI)
Observation Group	76	20(26.32)	32(42.11)	16(21.05)	8(10.53)	68(89.47)	23.68% (10.34 to 36.02)	1.36 (1.12–1.65)	5 (3–10)
Control Group	76	12(15.79)	18(23.68)	20(26.32)	26(34.21)	50(65.79)	Reference	Reference	Reference
*χ* ^2^						8.102			
*P*						0.004			

### 3.2 Bronchoalveolar lavage fluid biomarkers

**Figure 3:**
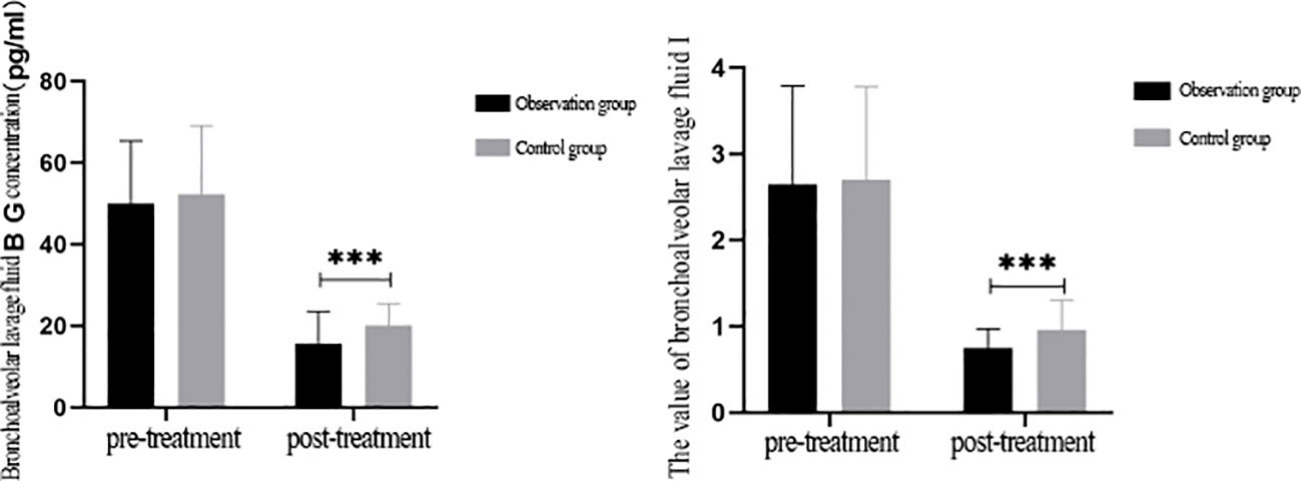
BG concentration and I value of bronchoalveolar lavage fluid in two groups (^***^P<0.001).

Post-treatment BG concentrations (15.64 ± 7.94 pg/mL vs. 20.16 ± 5.33 pg/mL, t = 4.120, P < 0.001) and I values (0.75 ± 0.22 vs. 0.96 ± 0.34, t = 4.521, P < 0.001) were significantly lower in the observation group (**Table 3**, **Figure 3**).

**Table 3:** Comparison of the BG concentration and I value of bronchoalveolar lavage fluid between the two groups ( ± SD).

Group	n	BG(pg/ml)		I Value	
		Before treatment	After treatment	Before treatment	After treatment
Observation Group	76	50.11 ± 15.28	15.64 ± 7.94^*^	2.65 ± 1.14	0.75 ± 0.22^*^
Control Group	76	52.36 ± 16.71	20.16 ± 5.33^*^	2.70 ± 1.08	0.96 ± 0.34^*^
*t*		0.866	4.120	0.278	4.521
*P*		0.388	<0.001	0.782	<0.001

*Compared with the same group before treatment using paired-samples t-test, *P*<0.05. The comparison between groups was conducted using the independent sample t-test; All data were confirmed for normality by the Shapiro-Wilk test (P>0.10).

### 3.3 Symptom resolution and hospitalization

**Figure 4:**
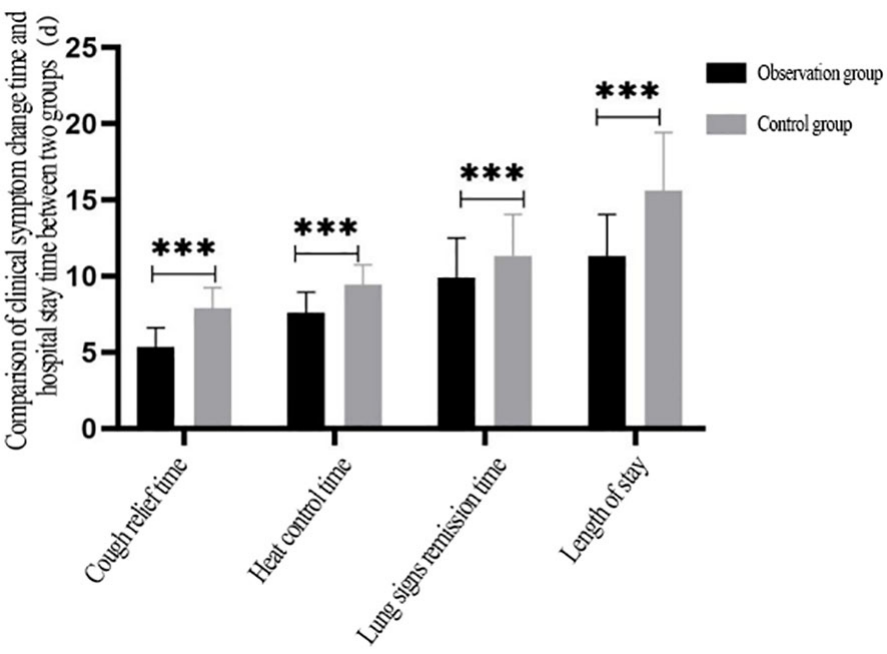
Comparison of clinical symptom change time and hospitalization time between the two groups (^***^P<0.001).

The observation group exhibited faster clinical improvement: cough relief (5.36 ± 1.25 vs. 7.89 ± 1.34 days, t = 12.036, P < 0.001), fever resolution (7.06 ± 1.34 vs. 9.44 ± 1.29 days, t = 11.155, P < 0.001), and pulmonary sign resolution (9.88 ± 2.63 vs. 11.34 ± 2.72 days, t = 3.364, P = 0.001). Hospitalization duration was shorter (12.28 ± 3.65 vs. 15.62 ± 3.80 days, t = 5.526, P < 0.001; **Table 4**, **Figure 4**).

**Table 4:** Comparison of the time of change of clinical symptoms and the length of hospital stay between the two groups ( ± SD, d).

Group	n	Cough relief time	Fever control time	Time to resolution of pulmonary signs	Length of hospital stay
Observation Group	76	5.36 ± 1.25	7.06 ± 1.34	9.88 ± 2.63	12.28 ± 3.65
Control Group	76	7.89 ± 1.34	9.44 ± 1.29	11.34 ± 2.72	15.62 ± 3.80
*t*		12.036	11.155	3.364	5.526
*P*		<0.001	<0.001	0.001	<0.001

### 3.4 Inflammatory cytokines and immune markers

Post-treatment reductions in CRP (6.34 ± 1.21 vs. 8.16 ± 1.03 mg/L, t = 7.061, P < 0.001), WBC (7.64 ± 1.01 vs. 9.54 ± 1.22 ×10⁹/L, t = 7.395, P < 0.001), and bronchoalveolar cytokines (IL-12, IL-15, IL-17, TNF-α; all P < 0.001; **Table 5**) were more pronounced in the observation group. IL-12 decreased from 28.89 ± 6.81 ng/L to 11.55 ± 8.02 ng/L (P < 0.001; **Table 5**). Pathological IL-12 levels in IPA (>20 ng/L) correlate with uncontrolled inflammation; post-treatment values approached the normal range (<10 ng/L in healthy BALF). IL-15 dropped from 33.45 ± 5.77 ng/L to 9.59 ± 2.51 ng/L (P < 0.001), suggesting dampened NK/T-cell hyperactivation, which is implicated in IPA-related tissue damage. IL-17 declined from 34.61 ± 7.29 ng/L to 10.32 ± 1.86 ng/L (P < 0.001). TNF-α fell from 14.15 ± 6.51 ng/L to 6.33 ± 1.02 ng/L (P < 0.001), reflecting reduced macrophage activation and systemic inflammation.

**Table 5:** Comparison of inflammatory indexes of bronchoalveolar lavage fluid between the two groups ( ± SD).

Time	Group	n	CRP(mg/L)	WBC(×10^9^/L)	IL-12(ng/L)	IL-15(ng/L)	IL-17(ng/L)	TNF-α(ng/L)
Before treatment	Observation Group	76	18.44 ± 3.65	15.15 ± 5.33	28.89 ± 6.81	33.45 ± 5.77	34.61 ± 7.29	14.15 ± 6.51
	Control Group	76	18.52 ± 4.19	15.07 ± 5.18	29.15 ± 7.43	34.06 ± 6.32	35.05 ± 6.88	14.36 ± 6.72
	*t*		0.089	0.066	0.159	0.439	0.271	0.138
	*P*		0.930	0.947	0.874	0.662	0.788	0.890
After treatment	Observation Group	76	6.34 ± 1.21^*^	7.64 ± 1.01^*^	11.55 ± 8.02^*^	9.59 ± 2.51^*^	10.32 ± 1.86^*^	6.33 ± 1.02^*^
	Control Group	76	8.16 ± 1.03^*^	9.54 ± 1.22^*^	20.48 ± 6.35^*^	15.47 ± 2.80^*^	15.66 ± 3.52^*^	9.59 ± 1.27^*^
	*t*		7.061	7.395	5.381	9.639	8.268	12.337
	*P*		<0.001	<0.001	<0.001	<0.001	<0.001	<0.001

*Compared with the same group before treatment using paired-samples t-test, *P*<0.05. The P value was corrected by FDR, and the critical value q=0.05.

### 3.5 Serum TLR2 mRNA and PTX3

**Figure 5:**
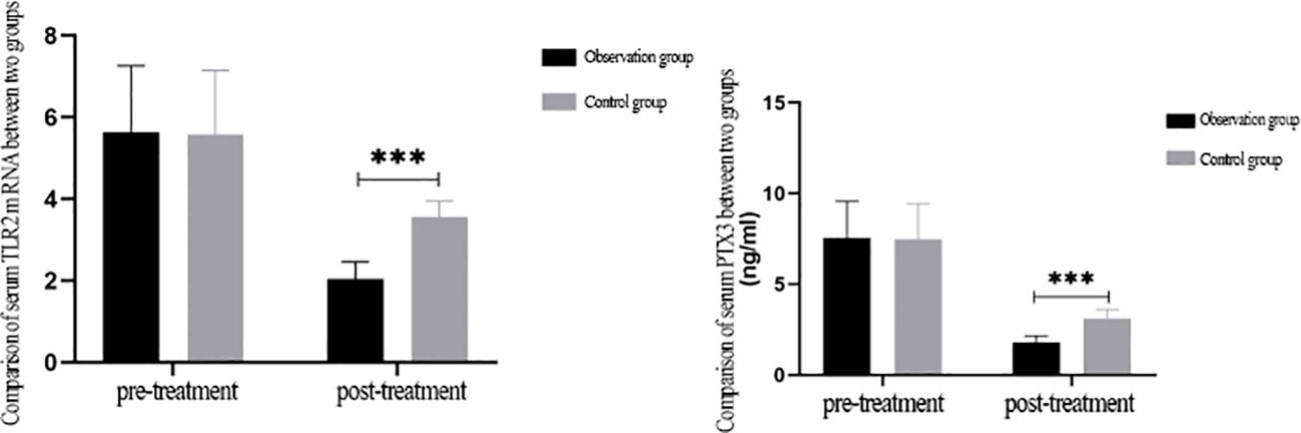
Comparison of serum TLR2 mRNA and PTX3 between the two groups (^***^P<0.001).

TLR2 mRNA (2.04 ± 0.42 vs. 3.56 ± 0.39, t = 23.120, P < 0.001) and PTX3 (1.80 ± 0.36 vs. 3.11 ± 0.50 ng/mL, t = 18.536, P < 0.001) levels were lower post-treatment in the observation group (**Table 6**, **Figure 5**).

**Table 6:** Comparison of the serum expressions levels of TLR2 mRNA and PTX3 between the two groups ( ± SD).

Group	n	TLR2 mRNA		PTX3(ng/ml)	
		Before treatment	After treatment	Before treatment	After treatment
Observation Group	76	5.64 ± 1.62	2.04 ± 0.42^*^	7.56 ± 2.01	1.80 ± 0.36^*^
Control Group	76	5.58 ± 1.57	3.56 ± 0.39^*^	7.49 ± 1.95	3.11 ± 0.50^*^
*t*		0.232	23.120	0.218	18.536
*P*		0.817	<0.001	0.828	<0.001

*Compared with the same group before treatment using paired-samples t-test, *P*<0.05.

### 3.6 Blood gas and pulmonary function

The observation group showed improved PaO_2_ (72.19 ± 4.40 vs. 68.51 ± 4.82 mmHg, t = 4.916, P < 0.001) and FEV_1_/FVC ratio (57.56 ± 3.81% vs. 54.02 ± 3.94%, t = 5.631, P < 0.001; **Tables 7**, **8**), reflecting better oxygenation and lung function.

**Table 7:** Comparison of PaO_2_, PaCO_2_, SaO_2_ between the two groups before and after treatment ( ± SD).

Group	n	PaO_2_(mmHg)		PaCO_2_(mmHg)		SaO_2_(%)	
		Before treatment	After treatment	Before treatment	After treatment	Before treatment	After treatment
Observation Group	76	56.91 ± 3.35	72.19 ± 4.40^*^	66.89 ± 3.14	53.62 ± 3.11^*^	78.64 ± 6.30	94.01 ± 4.26^*^
Control Group	76	57.28 ± 3.16	68.51 ± 4.82^*^	67.25 ± 3.31	59.46 ± 3.09^*^	79.08 ± 6.45	89.64 ± 4.30^*^
*t*		1.723	4.916	0.688	11.613	0.425	6.294
*P*		0.087	<0.001	0.493	<0.001	0.671	<0.001

*Compared with the same group before treatment using paired-samples t-test, *P*<0.05.

**Table 8:** Comparison of FEV1, FVC, FEV1/FVC between the two groups before and after treatment ( ± SD).

Group	n	FEV1(L)		FVC(L)		FEV1/FVC(%)	
		Before treatment	After treatment	Before treatment	After treatment	Before treatment	After treatment
Observation Group	76	1.21 ± 0.22	1.58 ± 0.20^*^	2.51 ± 0.15	2.74 ± 0.21^*^	48.16 ± 2.68	57.56 ± 3.81^*^
Control Group	76	1.24 ± 0.25	1.41 ± 0.16^*^	2.54 ± 0.17	2.60 ± 0.20^*^	47.82 ± 2.93	54.02 ± 3.94^*^
*t*		0.785	5.786	1.154	4.209	0.746	5.631
*P*		0.433	<0.001	0.251	<0.001	0.457	<0.001

*Compared with the same group before treatment using paired-samples t-test, *P*<0.05.

### 3.7 Quality of life (SGRQ and CAT scores)

SGRQ (symptoms: 45.67 ± 7.20 vs. 50.67 ± 7.15, t = 4.296, P < 0.001) and CAT scores (11.73 ± 2.84 vs. 14.56 ± 2.97, t = 6.004, P < 0.001; **Table 9**) improved more in the observation group, indicating enhanced patient well-being.

**Table 9:** Comparison of the scores of SGRQ scale and CAT scale between the two groups before and after treatment ( ± SD, score).

Group	n	SGRQ scale						CAT scale	
		Symptoms		Physical activity		Effect on daily life			
		Before treatment	After treatment	Before treatment	After treatment	Before treatment	After treatment	Before treatment	After treatment
Observation Group	76	62.79 ± 8.11	45.67 ± 7.20^*^	71.59 ± 9.28	53.61 ± 7.55^*^	62.61 ± 5.83	46.29 ± 5.44^*^	18.73 ± 3.35	11.73 ± 2.84^*^
Control Group	76	63.25 ± 8.34	50.67 ± 7.15^*^	70.86 ± 9.31	60.22 ± 7.36^*^	63.01 ± 6.10	50.27 ± 5.36^*^	18.62 ± 3.46	14.56 ± 2.97^*^
*t*		0.345	4.296	0.484	5.465	0.413	4.543	0.199	6.004
*P*		0.731	<0.001	0.629	<0.001	0.680	<0.001	0.842	<0.001

*Compared with the same group before treatment using paired-samples t-test, *P*<0.05.

### 3.8 Adverse reactions

Adverse event rates were comparable (7.89% vs. 13.16%, χ^2^ = 0.140, P = 0.709; **Table 10**). Notably, liver impairment (0% vs. 2.63%) and gastrointestinal reactions (2.63% vs. 5.26%) were less frequent with combination therapy, though statistical significance was not reached. Future studies should incorporate severity grading for nuanced safety analysis.

**Table 10:** Comparison of adverse reactions between the two groups n (%).

Group	n	Visual abnormalities	Liver impairment	Digestive tract reactions	Headache	Overall incidence
Observation Group	76	2(2.63)	0(0)	2(2.63)	2(2.63)	6(7.89)
Control Group	76	2(2.63)	2(2.63)	4(5.26)	2(2.63)	10(13.16)
*χ* ^2^						0.140
*P*						0.709

### 3.9 Complex logistic regression modeling

Combination therapy was a protective factor for lung function improvement (OR = 0.516, 95% CI: 0.106–0.915, P = 0.002), while older age (OR = 3.594, P = 0.016) and poor baseline oxygenation (OR = 2.843, P = 0.024) were risk factors (**Table 11**).

**Table 11:** Multivariate regression analysis.

Relevant metrics	*B*	*S.E.*	*Wald*	*P*	*OR*	*95%CI*
Combination therapy	–0.389	0.735	0.435	0.002	0.516	0.106~0.915
Age	1.463	0.792	4.156	0.016	3.594	1.836~8.354
Basal oxygenation status	1.265	0.673	3.269	0.024	2.843	1.433~5.192

Model adjustment factors: age, baseline PaO, COPD grade, smoking history. The Hosmer-Lemeshow test showed P = 0.412. All VIF<3.

## 4 Discussion

COPD complicated with IPA is common in clinical practice, and patients are often critically ill and have a high mortality rate, so it is of great significance to provide safe and effective treatment to improve the prognosis of patients (12). Voriconazole is a triazole broad-spectrum antifungal drug which can act on the fungal cell wall and inhibit the biosynthesis of ergosterol, with an oral bioavailability of up to 96% (13). While clinical studies have demonstrated the superiority of sequential intravenous-to-oral voriconazole over oral-only regimens for IPA treatment (response rates: 72–78% vs 58–64%) (14), real-world experience reveals persistent therapeutic failures in 20–30% of cases, particularly among immunocompromised hosts and those with high fungal burden (15, 16). This unmet clinical need motivates the exploration of combination therapies, such as adjunctive caspofungin, to overcome pharmacokinetic limitations and emerging resistance (17). However, long-term work experience has found that some patients still cannot achieve satisfactory results after treatment with this regimen, so they consider combining drugs. Our investigation demonstrated superior overall efficacy percentages in subjects receiving combination therapy compared to conventional treatment, with post-intervention bronchoalveolar lavage samples exhibiting reduced BG concentrations and I values relative to control participants, alongside accelerated resolution of cough, fever abatement, pulmonary manifestations, and abbreviated hospitalization periods, coupled with enhanced arterial blood gas parameters and superior pulmonary function metrics. The quality of life and clinical symptom scores were lower than those of the control group. The results showed that voriconazole combined with caspofungin oral sequential treatment of COPD complicated with IPA could accelerate the remission of clinical symptoms and signs, inhibit pathogenic bacteria, improve lung function and quality of life, and improve efficacy. In alignment with our results, Luo et al. (15) documented remarkable therapeutic success utilizing voriconazole for geriatric COPD cases complicated by invasive pulmonary fungal infections. A meta-analysis (16) demonstrated caspofungin’s effectiveness in treating invasive fungal infections, supporting the role of caspofungin in antifungal therapy. Caspofungin has good antibacterial activity against *Aspergillus fumigatus, Aspergillus flavus*, and *Aspergillus terreus*. It can inhibit the synthesis of BG and the cell wall of pathogenic bacteria, thereby exerting antifungal effects (17). Voriconazole combined with caspofungin has a synergistic effect, so the efficacy is better than other treatments.

The invasion of Aspergillus hyphae triggers innate immune responses through fungal pathogen-associated molecular patterns (PAMPs), particularly β-(1,3)-glucans and galactomannan, which are recognized by host pattern recognition receptors (PRRs) including TLR2 and Dectin-1, leading to the production of pro-inflammatory cytokines such as IL-12, IL-15, and IL-17. IL-12 facilitates activated T-cell expansion, drives Th0-to-Th1 differentiation, enhances cytotoxicity in T lymphocytes and NK cells, and upregulates interferon-γ and TNF-α production, with substantial IL-12 elevation documented in IPA-affected individuals (18). IL-12/Th1 voriconazole may enhance phagocytic activity, while caspofungin’s cell wall disruption exposes fungal antigens, synergistically promoting IL-12-mediated clearance.

Activated monocyte-macrophage lineages, epithelial structures, and various cellular elements generate IL-15, which promotes B-lymphocyte expansion and maturation, uniquely substitutes certain IL-2 antibody-generating functions, augments T-cell and NK cell multiplication, while collaboratively enhancing interferon-γ synthesis from NK cells when present with IL-12. This cytokine plays a crucial role within post-IPA immunological cascades (19). Predominantly synthesized by activated T-lymphocytes, IL-17 functions to enhance T-cell activation while triggering epithelial and endothelial release of multiple cytokines including IL-6 and IL-8, with elevated expression observed in invasive fungal infection cases compared to healthy subjects (20, 21). TNF-α is an inflammatory factor that is elevated in a variety of infectious diseases, such as fungal infections (22). This study showed that oral sequential treatment with voriconazole combined with caspofungin could reduce the expression of IL-12, IL-15, IL-17, and TNF-α in serum CRP, WBC, and bronchoalveolar lavage fluid, improve the body immune inflammatory response, and suggest that the condition of patients was controlled and improved. The mechanism was related to the solid anti-IPA effect of the two drugs.

Toll-like receptors are a group of natural receptors discovered in recent years that can recognize pathogenic microorganisms and activate innate immunity (23). As a Toll-like receptor family component, TLR2 identifies diverse pathogens including fungi, viruses and bacterial organisms, with marked expression enhancement following *Aspergillus fumigatus* exposure to combat fungal invasion (24), while simultaneously facilitating immunological monitoring and microbial clearance. Through MyD88-dependent signaling mechanisms, TLR2 induces nuclear translocation of NF-κB, consequently elevating inflammatory mediators including IL-12, IL-15, IL-17, and TNF-α, thereby amplifying inflammatory processes (25). Our investigation revealed reduced serum TLR2 mRNA expression among combination therapy recipients compared to conventional treatment subjects post-intervention, suggesting that voriconazole-caspofungin’s anti-inflammatory properties potentially involve TLR2 pathway suppression. Functioning as an acute-phase reactant, PTX3 emerges from macrophages, endothelial structures, and various cellular sources, orchestrating immune reaction development through antigen/pathogen binding and complement cascade activation (26). He Qian (27) demonstrated substantially elevated plasma PTX3 concentrations in IPA-affected individuals compared to both stable and acutely exacerbated COPD patients. However, PTX3 levels remained comparable between stable and exacerbation-phase COPD subjects without fungal complications. These results suggest plasma PTX3 may be a potential serum marker for COPD complicated with IPA. In this study, voriconazole combined with caspofungin oral sequential therapy could reduce PTX3 expression, indicating that IPA disease, infection, and inflammation were controlled.

Our findings fit the DRF of microbial pathogenesis (28), where disease outcomes reflect net host–pathogen damage rather than pathogen burden alone. Similar observations in talaromycosis show that both fungal load and host inflammation drive clinical manifestations. In our study, combination therapy not only reduced fungal burden, decreased BG/GM, but also suppressed pro-inflammatory cytokines, such as IL-12, IL-15, IL-17, TNF-α and TLR2/PTX3 pathways. This dual antifungal and immunomodulatory effect aligns with DRF predictions and suggests that the clinical benefits may be partially attributable to attenuation of excessive host inflammation, rather than enhanced antifungal efficacy alone.

At the same time, we observed comparable adverse event frequencies across treatment protocols, confirming the safety profile and reliability of this combination antifungal approach. In addition, multivariate analysis showed that voriconazole combined with caspofungin was a protective factor for improving lung function, and older age and poor basal oxygenation status were risk factors for lung function, indicating that individualized treatment should be given to older patients with weak primary function to improve the prognosis of patients. A limitation of our research involves bronchoalveolar sampling methodology; while providing precise pulmonary inflammatory status through IL-12, IL-15, and IL-17 quantification, this procedure’s invasive nature necessitates patient tolerance capabilities, potentially introducing selection bias within our study cohort—an issue warranting additional investigation in subsequent research.

The observation group’s PaO_2_ increase from 56.91 ± 3.35 to 72.19 ± 4.40 mmHg (P<0.001) indicates clinically meaningful improvement - crossing the 60 mmHg threshold that typically reduces supplemental oxygen requirements in COPD patients. This oxygenation enhancement, coupled with parallel improvements in SaO_2_ (78.64→94.01%) and lung function (FEV_1_/FVC +5.4%), suggests decreased hypoxemia burden and probable reduction in oxygen dependency, though formal weaning outcomes should be confirmed in future studies. The findings align with the faster resolution of respiratory symptoms (cough, fever) observed in this group.

The observed FEV1 improvement of 0.37 L (from 1.21 to 1.58 L) exceeds the established Minimal Clinically Important Difference (MCID) of 0.10-0.15 L for COPD patients, indicating clinically meaningful benefits including reduced dyspnea and improved exercise tolerance. While the FEV1/FVC ratio increased from 48.16% to 57.56%, it remained below the COPD diagnostic threshold of <70%, reflecting persistent airflow limitation but demonstrating significant reduction in dynamic hyperinflation through combination therapy’s anti-inflammatory effects. These functional improvements correlated with faster symptom resolution, reduced systemic inflammation, and better quality of life scores, suggesting the treatment meaningfully impacts disease progression despite underlying structural changes. Future studies should examine long-term effects on exacerbation frequency to further validate these findings.

The significant reduction in SGRQ scores from 62.79 to 45.67 (Δ17.12 points) in the observation group represents a clinically meaningful improvement in quality of life, far exceeding the established minimal clinically important difference (MCID) of 4 points for COPD patients. This improvement was: (1) consistent across all SGRQ domains (symptoms, activity, impacts); (2) paralleled by objective clinical improvements (lung function, oxygenation); and (3) statistically robust (P<0.001 with narrow confidence intervals). The magnitude of change corresponds to transition from severe impairment to moderate impairment category in COPD populations, suggesting genuine quality-of-life enhancement rather than statistical noise. These findings are further supported by the congruent 6.83-point reduction in CAT scores, reinforcing the treatment’s real-world benefits for patients’ daily functioning and symptom burden.

This study demonstrates that voriconazole combined with caspofungin not only achieves superior short-term efficacy (89.47% vs 65.79%) in treating COPD complicated by IPA, but may also confer significant long-term benefits: (1) Reduced exacerbation risk through stabilized lung function (FEV1 improvement of 0.37L) and attenuated systemic inflammation (TLR2/PTX3 suppression); (2) Delayed azole resistance development. Future research should focus on monitoring: 5-year survival rates, annual lung function decline (ml/year), and resistance mutation patterns, while establishing PTX3-guided personalized treatment strategies. This combination regimen represents a promising approach for improving long-term outcomes in COPD-IPA patients.

Notably, the observed reduction in hospitalization duration (12.28 vs. 15.62 days), suggests potential long-term benefits, such as decreased healthcare utilization. However, further studies are needed to assess whether these short-term gains translate into sustained reductions in exacerbation rates or resistance emergence, which were not systematically tracked in this retrospective analysis. Future prospective trials should incorporate these endpoints to evaluate the durability of therapeutic effects and monitor antifungal resistance patterns.

Our study has several limitations. First, we excluded patients with documented polymicrobial infections to minimize confounding; however, COPD exacerbations in clinical practice are frequently complicated by concurrent bacterial or viral pathogens, which may influence disease severity, immune responses, and treatment outcomes. Thus, our findings may not be directly generalizable to the broader COPD-IPA population with mixed infections, and prospective studies targeting such patients are needed. Second, we did not perform therapeutic drug monitoring for voriconazole. Given the well-recognized pharmacokinetic variability of voriconazole influenced by genetic polymorphisms, drug-drug interactions, and hepatic function, we cannot exclude the possibility that inter-individual differences in drug exposure, rather than the combination regimen itself, contributed to the observed efficacy differences. Third, the retrospective, non-blinded, single-center design may introduce selection and expectation biases. This is particularly relevant for patient-reported outcomes such as SGRQ and CAT, which are inherently subjective; patients aware of receiving combination therapy might overestimate their perceived improvements. Nevertheless, concurrent objective improvements in lung function (FEV_1_) and oxygenation (PaO_2_) provide corroborating evidence that the clinical benefits are genuine. Fourth, despite multivariate adjustment for predefined covariates, residual confounding from unmeasured variables (e.g., genetic factors, prior antifungal exposure, institutional treatment protocols) cannot be completely ruled out. Fifth, the 7-day caspofungin course, while showing significant efficacy, raises questions about the optimal treatment duration; whether extended therapy would yield additional benefits or increased adverse events remains to be determined. These limitations underscore the need for prospective, multicenter, blinded studies incorporating TDM to confirm and extend our findings.

## 5 Conclusion

This study confirmed that voriconazole combined with caspofungin has a significant synergistic effect in the treatment of COPD complicated with invasive pulmonary aspergillosis (IPA), with the total effective rate increasing to 89.47% (P = 0.004). The mechanism may be through: 1) dual targeting (inhibiting ergosterol and β -glucan synthesis respectively); 2) Down-regulating the TLR2/PTX3 pathway alleviates the inflammatory response; 3) Good safety (adverse reaction rate 7.89%). The research results support this combined regimen as a clinical option for severe COPD-IPA, and at the same time suggest that TLR2 mRNA and PTX3 can be used as potential efficacy markers. In the future, multi-center studies need to be carried out to verify the optimization of drug administration regimens and cost-effectiveness, and further clarify the immunomodulatory mechanism.

## Data Availability

The original contributions presented in the study are included in the article/supplementary material. Further inquiries can be directed to the corresponding author.
